# Design and Analysis of a Reconfigurable Gilbert Mixer for Software-Defined Radios

**DOI:** 10.3390/s21082711

**Published:** 2021-04-12

**Authors:** Shilpa Mehta, Xue-Jun Li, Massimo Donelli

**Affiliations:** 1 Department of Electrical and Electronic Engineering, Auckland University of Technology, Auckland 1010, New Zealand; xuejun.li@aut.ac.nz; 2 Department of Information Engineering and Computer Science, University of Trento, 38100 Trento, Italy; massimo.donelli@unitn.it

**Keywords:** software-defined radio, mixer, image-rejection ratio, impedance matching

## Abstract

A reconfigurable $g_{m}$-boosted, image-rejected downconversion mixer is presented in this paper using the SiGe 8 HP technology. The proposed mixer operates within 0.9–13.5 GHz that is suitable for software-defined radio applications. The conversion mixer comprises of resistive biased radio frequency (RF) section, double balanced Gilbert cell mixer core sections divided as per I and Q stages for image-rejection purpose, inductively peaked $g_{m}$-boosting section and tunable filter section, respectively. In comparison to previous works in the scientific literature, the design shows enhanced conversion gain (CG), noise figure (NF), and image-rejection ratio (IRR). For the entire band of operation, the mixer attains a good return loss |S11| of <−10 dB. Additionally, the design accomplishes an excellent CG of 22 dB, NF of 2.5 dB, and an image-rejection ratio of 30.2 dB at maximum frequency. Finally, a third-order intercept point (IP3) of −3.28 dBm and 1 dB compression point (CP1) of −13 dBm, respectively, shows moderate linearity performance.

## 1. Introduction

Wireless communications have become increasingly popular due to the wide range of potential applications. In recent years, this industry has experienced tremendous development leading to many wireless standards. Therefore, it is desired to have a radio front end that is capable of handling multiple standards and applications [1,2,3]. For developing such receiver circuits, especially within high-frequency bands, proper radio frequency (RF) and baseband blocks must be available to perform downconversion operation. Ideally, distinct radio front ends can be used for different standards and applications. However, this is not possible due to frequency sensitivity [4,5]. Hence, RF-front-ends are compatible with distinct standards operating at specific frequencies. In addition, one must develop advanced systems with modern blocks with the emergence of the latest wireless standards. Reconfigurable blocks can be reused to accommodate multiple wireless standards, and thus lower development time and cost [6]. Software-defined radios (SDRs) offer this flexibility by allowing multiple band operations inside a single circuitry [7,8], whereas cognitive radios (CRs) take care of spectrum crowding and congestion hurdles [9,10]. Although most of the research is based on single-band cognitive radio, multiband cognitive radio has greater potential in the efficient implementation of cognitive networks. It is expected that multiband cognitive radios improve the throughput and lower handoff frequency for better channel maintenance. However, wideband front end and access to multiband spectrum present several challenges [11]. Thus, the best is to have CR with SDR features, which can sense the electromagnetic spectrum environment, tracking and responding to the variations and findings in a smart way. Moreover, the cost and power consumption are significant while talking about reconfigurable receiver architectures [12]. Earlier receivers were using analog-to-digital converters. Signal processing is carried out in the digital domain that consumes high power and attains inaccurate RF signals, which limits the use of SDRs. Thus, to overcome that, mixers must be used [13].

Mixers are used for frequency conversion purposes. Upon mixing, the frequency of the output signal is in the form of a sum or difference of those of the input signals. Within the receiver, mixers can perform frequency downconversion to shift RF to IF [14]. For good performance with low NF, high linearity, polarity switching through local oscillator (LO) input is required. Such a mixer will have radio frequency (RF) signals divided into in-phase and out-of-phase parts; the conversion switch operated by LO signal can alternatively choose in-phase and out-of-phase signals. Ideally, mixers will introduce the minimum amount of noise and have good linearity [15]. Moreover, they should be independent of LO amplitude and intermodulation products. However, practical mixers have the following limitations: non-negligible NF, limited CG, and linearity [16]. Mixers are broadly classified as passive mixers and active mixers. Passive mixers introduce signal attenuation and mixing is achieved through passive switches. Therefore, the switches are turned on and off depending on the LO signal, which is compared to a reference voltage and mixing is achieved through the multiplication operation of RF and LO frequency signals (in terms of square wave or sinewave). These mixers are widely used because of their simplicity, zero power consumption, high IP3, and good NF at the expense of port isolation. However, its main drawback is the high LO power requirement [14]. In contrast to passive mixers, active mixers can provide high CG, good port isolation, low NF, and low LO power requirements. However, it is difficult for them to achieve good linearity. Thus, based on the advantages, active mixers are preferred over passive mixers. Among active mixers, Gilbert mixer is the most commonly used architecture that follows a double balanced structure. This configuration shows high isolation [17]. The mixer also attains high performance in terms of CG, NF at the expense of linearity [18]. The systematic approach can be used further to improve the overall performance that estimates the proper width-to length (W/L) ratio to attain the required design specifications. This approach shows a good trade-off among different performance parameters [19].

For SDR, a single mixer operating within a wideband would be able to convert multiple RF signals to a single IF signal, reducing the design complexity and overall cost [20]. In general, the overall performance of the receiver depends on various metrics such as dynamic range, IP3, NF, CG, IRR, filtering, signal-noise ratio, and spurious-free dynamic range, respectively. Nevertheless, there are no industrial standards defined for SDRs, but they should be able to attain at least high CG, high IRR, and low NF, respectively [21]. Additionally, SDR mixers should be able to provide linear operation while maintaining stability. However, there are a limited number of SDR mixers that provide good reconfiguration, cover a wide bandwidth, and attain high IP3 due to additional circuitry requirements [22,23]. The design complexity should also be low while consuming low power to prolong the battery lifespan [24].

Reconfigurable SDR mixers can be either switchable or tunable. In case of switchable SDR mixers, reconfiguration is possible using switches along with other components such as inductors, transformers. However, tunable mixers use tunable resonators for reconfiguration. With strict bandwidth requirements, analog or discrete tuning can be opted. Numerous techniques exist for possible tuning of operating frequency, among which typical techniques include transmission line-based designs with limited frequency of operation [25,26], transformer dependent inductors with variable frequency of operation [27], analog bias tuning with limited tuning range, array arranged filters, charge domain discrete-time filters [28], and polyphase networks [29], respectively. Tuning is important to lower the RF front-end section area for multifunctional, multiple frequency, and multistandard applications. Moreover, easy reconfiguration will be done as per the parameters’ necessity with respect to the standards, band of operation and overall performance. Thus, it is expected to have a single tunable filter that can replace the large and expensive filter banks, has a wide operating band, and can be easily optimized as per the standards, band or requirements. Moreover, it should cover a small chip area and consumes less power [30]. Hence, for proper reconfiguration to occur, it is important to have perfect tuning based on the above-mentioned approaches.

Gilbert mixer is quite common among SDR receiver architectures as they can provide high bandwidth and broadband performance without degradation in the performance parameters [31]. Several mixer designs have been discussed in [32,33,34,35,36,37]. As in [38], improved Gilbert mixer design is proposed that attains reasonable CG and high port isolation while operating within a wideband. Likewise, in [39] a reconfigurable Gilbert mixer is proposed that employs a passive switchable network (a combination of switched capacitor and inductors) for tuning purposes, resulting in a highly flexible design. The design attained high CG, moderate IP3 at the expense of NF. To overcome the issues within the above-mentioned designs, a joint LNA + mixer-based design can be used. The proposed design maintains a small chip size and consumes less power [40]. Noise-cancelling plays an important role for such a front-end topology. However, it depends on the proper metric matching based on I/Q mixer topology. Another common approach is to use partial noise-cancelling with a folding mixer architecture with no current reuse, specifically for wideband and low power operation. In this case, *g*_m_-boosting is the better option, which is independent of performance metrics matching as used in LNA section. Finally, in [5], a *g*_m_-boosted technique is employed in the RF stage of the mixer that helps in CG improvement with low power consumption.

Based on the above-mentioned approaches, a novel Gilbert mixer is proposed that employs *g*_m_-boosting technique with high image rejection. For tuning purposes, the ninth-order tunable resonator has been developed using varactors and inductors, where the varactors are responsible for providing tuning behavior within the mixer. In this paper, we discuss the design and analysis of the mixer achieving tunable input-output matching from 0.9 GHz to 13.5 GHz. The rest of the paper is organized as follows: Section 2 focuses on motivation. Section 3 presents the mixer design, followed by the analyses of the proposed mixer in Section 4. Section 5 discusses about the simulation results and the layout of the proposed mixer. The proposed mixer’s reliability performance is analyzed using Relxpert software as discussed in Section 6 and finally Section 7 concludes the paper.

## 2. Motivation

**Figure 1:**
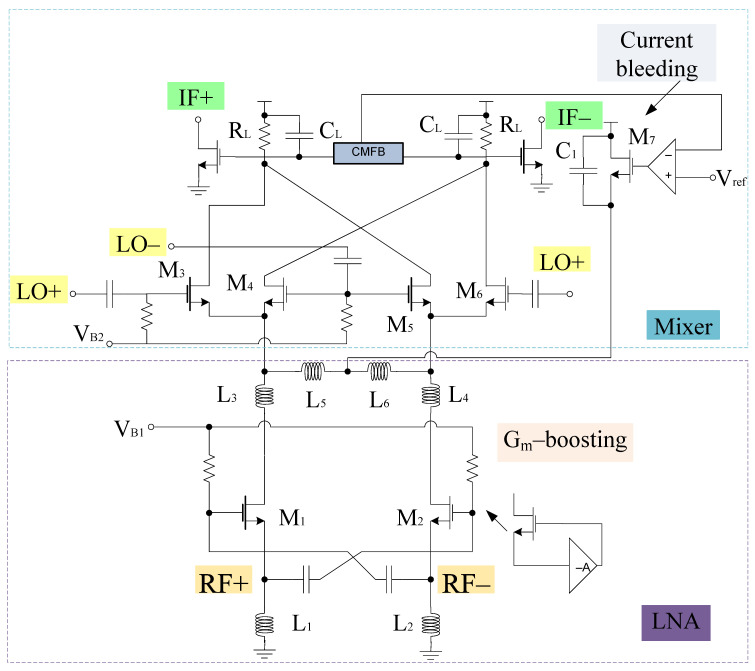
Merged LNA and mixer.

**Figure 2:**
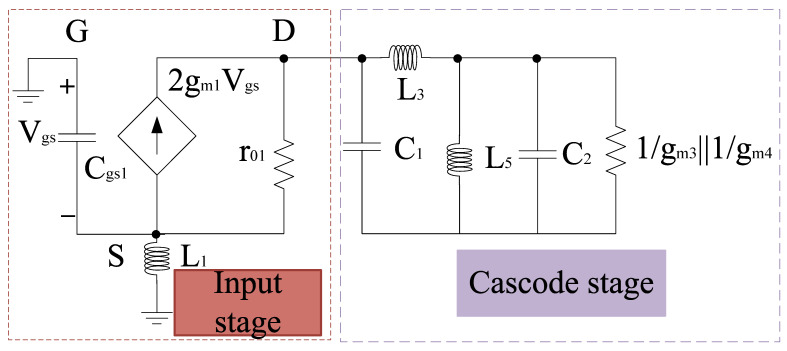
Small signal model.

A merged LNA and mixer circuitry is shown in Figure 1. It consists of *g*_m_-boosting section, and a source inductor at the input end. At the output end capacitors and inductors (ignoring parasitic resistance) are connected to the LNA section. LNA and mixer employ *g*_m_-boosting and current bleeding techniques, which makes the design capable of attaining high CG, low NF, and low power consumption. The design also employs the current peaking approach for wideband operation [40]. Therefore, the input and output impedance can be expressed using a small signal model as shown in Figure 2.

Input impedance, *Z*_in_ can be expressed as:

(1) $$Z_{in}=\frac{sL_{1}}{1+\left(G_{m1}+sC_{gs1}\right)sL_{1}}$$

where *C*_gs1_ refers to the gate to source capacitance at the input end. *G*_m_-boosting helps in overall gain improvement as it improves the overall transconductance in the stage to which it is connected by the factor (−A) as shown in Figure 1. Upon analysis, it can be expressed as

(2) $$G_{m1}=\frac{r_{01}+Z_{L}}{1+2g_{m1}r_{01}}$$

where *G*_m1_ refers to *g*_m_-boosting term; *g*_m1_, *r*_01_, and *Z*_L_ refer to the transconductance, transistor impedance, and load impedance, respectively. Similarly, the output impedance, *Z*_L_ can be expressed as:

(3) $$Z_{L}=\frac{1}{sC_{1}}||[sL_{3}+\left(\frac{1}{sC_{2}}\right)||sL_{5}||(\frac{1}{g_{m3}}\left|\right|\frac{1}{g_{m4}})]$$

where *C*_1_, *C*_2_ refers to the interstage parasitic capacitance. *g*_m3_ and *g*_m4_ refer to the transconductance of *M*_3_ and *M*_4_ transistors. As per the input network, the resonant frequency depends on *C*_gs1_ and *L*_1_. The resonant frequency at which the input impedance, *Z*_in_ will be real can be defined as

(4) $$f_{0}=\frac{1}{2\pi \sqrt{L_{1}C_{gs1}}}$$

Therefore, to attain the tunable frequency, *f*_0_ and input impedance, it is desired for *Z*_in_ to use the tunable filter components. This will enhance not only the overall chip area and power consumption but also the performance metrics such as noise figure. In this paper, we propose an active mixer with a tunable filter, comprising of variable capacitors and inductors. Therefore, in order to attain the minimum return loss, S_11_ at each centered frequency, the input impedance can be varied with the variation in the current. To improve the performance of the circuitry, the design also employs the *g*_m_-boosting technique.

## 3. Proposed Mixer Topology

**Figure 3:**
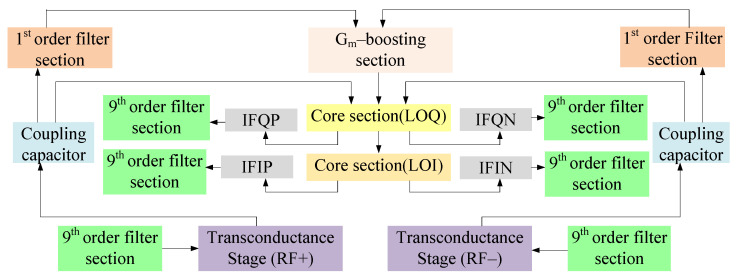
Block diagram of the proposed mixer.

Figure 3 shows the block representation of the proposed mixer with different stages. Starting from the bottom, Stage I refers to the transconductance stage that follows a common source configuration. Stage II refers to the core section categorized into LOI (local oscillator in-phase) and LOQ (local oscillator out-of-phase) stages where input signals are in 90 degree phase shift with respect to each other. For coupling, transconductance and core stages, coupling capacitors have been used. The third stage refers to the *g*_m_-boosting stage and finally, stage IV discusses the filters, i.e., first-order filter for avoiding leakage from the power supply and the ninth-order tunable filter for impedance matching at RF and IF stages. For a better understanding of the proposed circuit topology, the design and analysis of all stages have been discussed as follows.

### 3.1. Tranconductance Stage

**Figure 4:**
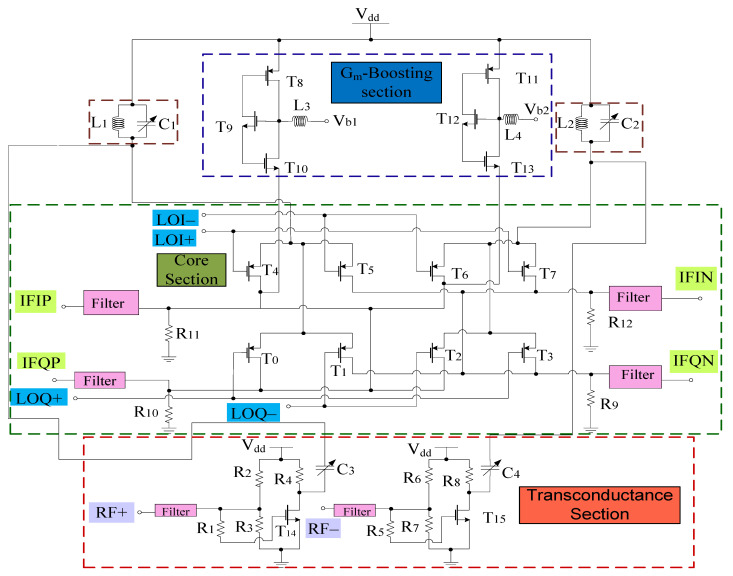
Proposed mixer.

**Figure 5:**
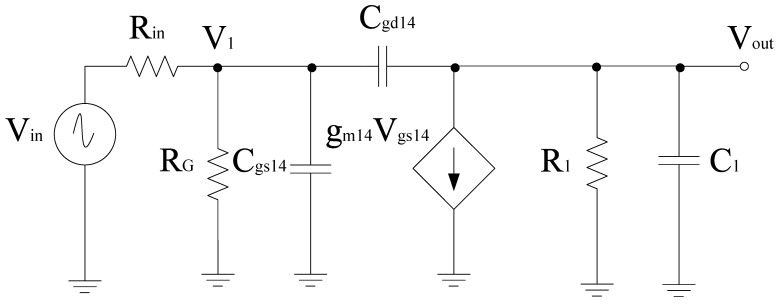
Equivalent small signal model for the RF+ stage.

Figure 4 shows the complete circuit diagram of the proposed mixer. Based on the design, the transconductance stage follows the common source configuration and is categorized into different sections, i.e., RF+ and RF− stage, respectively. Both RF+ and RF− stages consist of resistors arranged in a shunt configuration with RF+ stage that consists of input resistors *R*_1_, *R*_2_, and *R*_3_. Similarly, RF− consists of input resistors *R*_5_, *R*_6_, and *R*_7_, respectively. *R*_1_ and *R*_5_ resistors are responsible for the overall input resistance of each stage and can alter the input voltage and hence the overall gain performance. *R*_4_ and *R*_8_ refer to the load resistors opted while keeping the desired drain current I_D_. Filters are also linked to the input end of these stages for impedance matching purposes. Transistor W/L ratio is in such a way that it satisfies the core stage transistor saturation region operating conditions. The input, output impedance, and gain expression of the RF stages can be obtained with the help of the equivalent small signal model. Figure 5 shows the small signal model while ignoring the filter whose analysis is discussed in the filter section. Therefore, for the RF+ stage *R*_in_ is the internal resistance, *R*_G_ refers to the gate resistance, which is the parallel combination of *R*_1_, *R*_2_, and *R*_3_ respectively. Input impedance of RF+ stage

(5) $$Z_{in}=R_{G}=R_{1}\left|\right|R_{2}\left|\right|R_{3}$$

Output impedance of RF+ stage

(6) $$Z_{out}=R_{1}\left|\right|\frac{1}{jwC_{1}}=\frac{R_{1}}{\left(R_{1}\right)\left(jwC_{1}\right)+1}=\frac{R_{1}}{\left(R_{1}\right)\left(sC_{1}\right)+1}$$

where *R*_1_ = *r*_ds_14__= *R*_4_, *sC*_1_ = *sC*_db14_+ *sC*_3_. Similarly, the input impedance of RF- stage can be represented as

(7) $$Z_{in}=R_{G}=R_{5}\left|\right|R_{6}\left|\right|R_{7}$$

Output impedance of RF− stage

(8) $$Z_{out}=R_{2}\left|\right|\frac{1}{jwC_{2}}=\frac{R_{2}}{\left(R_{2}\right)\left(jwC_{2}\right)+1}=\frac{R_{1}}{\left(R_{2}\right)\left(sC_{2}\right)+1}$$

where *R*_2_ = *r*_ds_15__= *R*_8_, *sC*_2_ = *sC*_db15_+ *sC*_4_. Therefore, to obtain the frequency response of the small signal circuit, nodal analysis can be done. The first term should be the node at which the currents are added. If node voltages are multiplied, it refers to all admittances being connected to a node. Next terms will have negative signs which are actually neighboring node voltages and each of these terms uses a multiplication operation on the connecting admittance. The final terms refer to the current sources having a positive sign that is considered only if current sources are flowing out of that node [41]. Based on this, we have

(9) $$V_{1}\left(G_{G}+sC_{gs14}+sC_{gd14}\right)-V_{in}G_{G}-V_{out}sC_{gd14}=0$$

(10) $$V_{\mathrm{out}}\left(G_{1}+sC_{1}+sC_{\mathrm{gd}14}\right)-V_{1}sC_{\mathrm{gd}14}+g_{m14}V_{\mathrm{gs}14}=0$$

As *V*_1_ = *V*_gs_, then from Equation (10), we get;

(11) $$V_{1}=\frac{V_{\mathrm{out}}\left(G_{1}+sC_{1}+sC_{\mathrm{gd}14}\right)}{-g_{m14}+sC_{\mathrm{gd}14}}$$

By substituting (11) in (9), we get;

(12) $$\begin{array}{c} V_{\mathrm{out}}[G_{1}G_{G}+s\left[G_{1}\left(C_{\mathrm{gs}14}+C_{\mathrm{gd}14}\right)+G_{G}\left(C_{\mathrm{gd}14}+C_{1}\right)+g_{m1}C_{\mathrm{gd}14}\right]+s^{2}[\left(C_{\mathrm{gs}14}+C_{\mathrm{gd}14}\right)\left(C_{1}+C_{\mathrm{gd}14}\right)\\ -C^{2}{}_{\mathrm{gd}14}]]=V_{\mathrm{in}}G_{G}\left(-g_{m14}+sC_{\mathrm{gd}14}\right) \end{array}$$

(13) $$\frac{V_{\mathrm{out}}}{V_{\mathrm{in}}}=\frac{-g_{m14}\left(1-s\frac{C_{\mathrm{gd}14}}{g_{m14}}\right)R_{1}}{1+sa+s^{2}b}$$

(14) $$a=R_{G}\left[C_{\mathrm{gs}14}+C_{\mathrm{gd}14}\left(1+g_{m14}R_{1}\right)\right]+R_{1}\left(C_{\mathrm{gd}14}+C_{1}\right)$$

*R*_in_ is not used and upon simplification and converting *G*_G_, *G*_1_ to *R*_G_, *R*_1_, we get;

(15) $$b=R_{G}R_{1}\left(C_{\mathrm{gd}14}C_{\mathrm{gs}14}+C_{\mathrm{gs}14}C_{1}+C_{\mathrm{gd}14}C_{1}\right)$$

If s = 0, the low frequency gain is obtained as mentioned below:

(16) $$A_{v}=-g_{m14}R_{1}$$

When the poles are real and

(17) $$w_{p1}<<w_{p2}$$

The denominator of Equation (13) becomes

(18) $$D\left(s\right)=\left(1+\frac{s}{w_{p1}}\right)\left(1+\frac{s}{w_{p2}}\right)=1+\frac{s}{w_{p1}}+\frac{s^{2}}{w_{p1}w_{p2}}$$

Comparing Equation (13) with Equation (18), we get

(19) $$w_{p1}=\frac{1}{a}$$

(20) $$w_{p2}=\frac{1}{bw_{p1}}$$

Similarly, for RF− stage, the simplified gain can be expressed as mentioned below:

(21) $$A_{v}=-g_{m15}R_{2}$$

### 3.2. Core Stages

**Figure 6:**
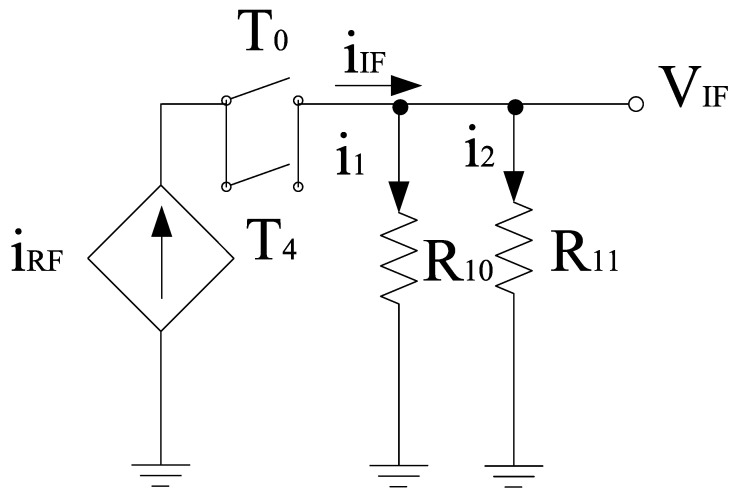
Equivalent small signal model for LO stage.

It is well known that the image signal is an unwanted input signal to the mixer. Its frequency will be above or below the local oscillator (LO) frequency by an amount which is equal to the IF frequency. Suppose if f_R1_ is the frequency of the desired input signal, then f_R2_ is for its image. Thus, both image and actual input signals mix with the LO and will downconvert to the same frequency. This is problematic for the mixer as both downconverted products interfere with each other as they exit at the IF port together. Thus, by using separate LO stages will overcome this problem and the outputs will be obtained at different IF stages. Based on this phenomenon, two core stages have been developed for the proposed mixer whose outputs will be obtained at different IF stages. The switching stages steered by LO inputs are classified as in-phase (I) and quadrature-phase (Q) stages for image-rejection purposes. Both LO stages contain p-type field effect transistors (FETs) for flicker noise reduction purpose, the transistors T_0_–T_3_,T_4_–T_7_ are the part of LOQ and LOI stages, respectively. Alternate transistors in each stage form a differential pair and operate alternatively when LO pulse is applied. Hence, differential outputs will be obtained and the current switch between outputs. The output current is directly proportional to the input current and the signal that is applied at the gate terminals. For determining the output voltage, the current flowing through load resistors is considered along with the load resistors itself. In the design, coupling capacitors are used to couple RF and LO stages. The small signal model for obtaining the output voltage with respect to the current obtained from the RF stage is shown in Figure 6. The model uses Kirchoff’s current law (KCL) for analysis. Therefore,

(22) $$i_{\mathrm{IF}}=i_{1}+i_{2}$$

(23) $$i_{\mathrm{IF}}=\frac{V_{\mathrm{IF}}}{R_{10}}+\frac{V_{\mathrm{IF}}}{R_{11}}$$

(24) $$i_{\mathrm{IF}}=V_{\mathrm{IF}}\left(\frac{1}{R_{10}}+\frac{1}{R_{11}}\right)$$

(25) $$i_{\mathrm{IF}}=V_{\mathrm{IF}}\left(\frac{R_{10}+R_{11}}{R_{10}R_{11}}\right)$$

(26) $$\frac{V_{\mathrm{IF}}}{i_{\mathrm{IF}}}=\frac{R_{10}R_{11}}{R_{10}+R_{11}}$$

### 3.3. Gm-Boosting Section

G_m_-boosting circuitry is connected to core stages, which is responsible for the transconductance improvement. Hence, the conversion gain will be enhanced while controlling the power consumption [5]. The proposed design employs peaking inductors at the gate of the transistors. These inductors resonate with the parasitic capacitances and are responsible for avoiding current leakage. The design also employs P-type FETs (T_8_ and T_11_) connected to V_dd_. However, (T_9_–T_12_) are N-type FETs. The design follows the stacking structure, where transistors T_9_ and T_12_ act as an amplifier that improves *g*_m_ and overall gain by a factor of (−A). Transistors T_10_ and T_13_ are responsible for delivering the current to the connecting stages. All transistors are operating in the saturation region. The drain current will start flowing in the core stages, the current obtained from this stage will bleed to transconductance stage. Hence, the current will be reused by the transistors T_14_ and T_15_, respectively. The equivalent circuit for the *g*_m_-boosting stage is shown in Figure 6.

When the transistors T_8_, T_10_, T_11_, and T_13_ are operating in the saturation region, the current flowing through that stage is given by:

(27) $$i_{\mathrm{GM}+}=I_{D8}+I_{D10}-\left(1+A\right)g_{m8}g_{m10}v_{\mathrm{RF}}$$

Similarly, for the other section

(28) $$i_{\mathrm{GM}-}=I_{D11}+I_{D13}-\left(1+A\right)g_{m11}g_{m13}v_{\mathrm{RF}}$$

The LO switches are considered ideal. Therefore, during the positive half of LO pulse, the output current will be positive; during the negative half cycle, the current obtained will be negative. The total current due to the half LOI stage is represented as:

(29) $$i_{0}=I_{D4}-I_{D5}=I_{D11}+I_{D13}-\left(1+A\right)g_{m11}g_{m13}v_{\mathrm{RF}}$$

This current is transferred to the LOQ stage, then the current within this stage will be due to LOI and the stage itself

(30) $$i_{1}=i_{0}+I_{D0}-I_{D1}$$

**Figure 7:**
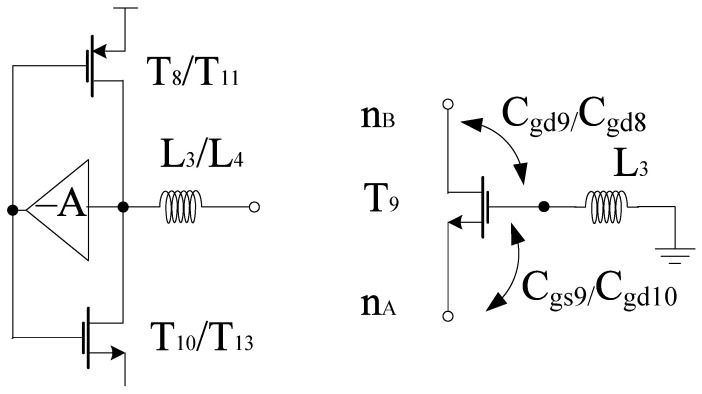
Gm stage model.

As the coupling capacitors connect the LO stages to the RF stage, the overall boosting can be observed in terms of CG, where the *g*_m_ stage is acting in parallel with the load at the IF end. Moreover, *g*_m_-boosting inductor L_3_ or L_4_ present within the design are responsible for gain improvement. The design analysis is explained as per the positive feedback theory whose model is shown in Figure 7 . For using this principle, the T_9_ signal paths are taken into consideration and output impedance has been ignored for simplicity. Thus, due to presence of L_3_ or L_4_ a non-zero impedance can be observed at the gate terminal of T_9_. The feedforward and feedback paths are considered using parasitic capacitances such as gate-source capacitance (Cgs) and the gate-drain capacitance (Cgd), respectively. Thus, gate-source voltage of T_9_ becomes *V*_g,T9_ while considering new signal paths. The next step is to calculate the open-loop voltage gain as per voltage-voltage feedback configuration. Hence, the voltage at the drain terminal is expressed as

(31) $$V_{nB}=-g_{m9}V_{\mathrm{gs}9}Z_{nB}=g_{m9}\left(V_{\mathrm{nA}}-\alpha V_{\mathrm{nA}}\right)Z_{\mathrm{nB}}$$

where *V*_gs9_, *g*_m9_ and *Z*_nB_ refers to the gate to source voltage of T_9_, transconductance of T_9_ and output impedance at node nB that includes the duplicate, respectively. where

(32) $$Z_{\mathrm{nB}}=Z^{\prime }{}_{\mathrm{nB}}\left|\right|\left(sL_{3}+\frac{1}{sC_{gd9}}\right)$$

(33) $$Z^{\prime }{}_{\mathrm{nB}}=\frac{1}{sC_{gd9}}\left|\right|\frac{1}{sC_{gd8}}\left|\right|\left(\frac{1}{g_{\mathrm{mLOI}}+g_{\mathrm{mLOQ}}}\right)$$

where *Z*^’^_nB_, α, *C*_gd9_ and *C*_gd8_ refer to the output impedance excluding the duplicate, voltage ratio from source to gate, parasitic capacitances for T_8_ and T_9_, respectively. Thus, the open-loop gain is represented as

(34) $$A_{0}=\left(1-\alpha \right)g_{m9}Z_{\mathrm{nB}}$$

where

(35) $$\alpha =\frac{sL_{3}\left|\right|\frac{1}{sC_{gd9}}\left|\right|\frac{1}{sC_{gd8}}}{(\frac{1}{sC_{gs9}}\left|\right|\frac{1}{sC_{gd10}})+sL_{3}\left|\right|\frac{1}{sC_{gd9}}}$$

Finally, the voltage gain (*A*_V0_) without the feedback inductor and the closed-loop voltage gain (*A*_Vf_) can be expressed as

(36) $$A_{v0}=\frac{V_{nB}}{V_{\mathrm{nA}}}{{|}_{\left(w/o\right)L_{3}}=g_{m9}Z_{\mathrm{nB}}|}_{\left(w/o\right)L_{3}}$$

(37) $$A_{\mathrm{vf}}=\frac{V_{\mathrm{nB}}}{V_{\mathrm{nA}}}{|}_{\left(w\right)L_{3}}=\frac{A_{0}}{1+\beta A_{0}}$$

where

(38) $$Z_{\mathrm{nB}}{|}_{\left(w/o\right)L_{3}}=Z^{\prime }{}_{\mathrm{nB}}\left|\right|\frac{1}{sC_{gd9}}$$

(39) $$\beta =\frac{sL_{3}}{(\frac{1}{sC_{gd9}}||\frac{1}{sC_{gd9}})+sL_{3}}$$

where β is the feedback factor from drain to gate Hence, it has been verified from the above equations that the gain has been boosted in the presence of the inductor.

### 3.4. Filter Section

**Figure 8:**
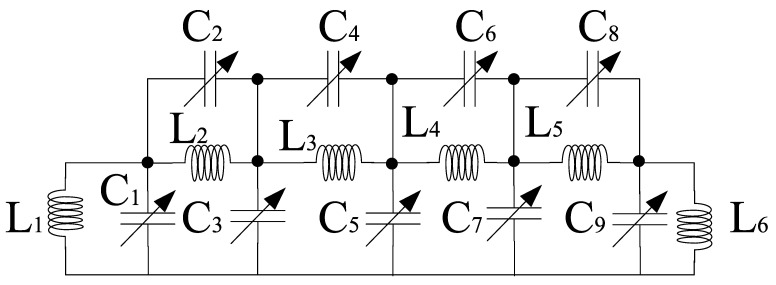
Ninth-order filter.

**Figure 9:**
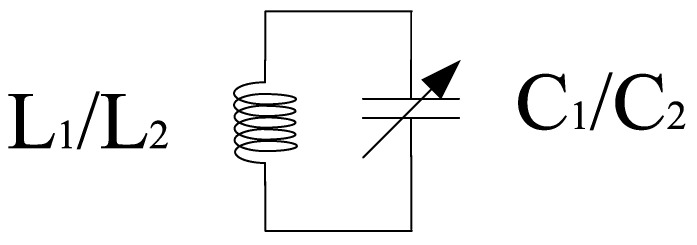
First-order filter.

Filters present at the input and output stages are responsible for impedance matching at various frequencies within a band. However, the ones near the core stage prevents signal leakage from the power supply. Various filters have been proposed and the most common techniques are transmission lines, transformer dependent programmable or spiral inductors, and dual-behavior resonator topology [42,43,44,45]. However, these filters are limited to some extent and may cover a large area. Therefore, off-chip filters can be used but easy integration is not possible and they are expensive as well. Hence, the most convenient approach is to develop an image-rejected mixer that employs different filters. We propose ninth-order band pass tunable filters for impedance matching purpose which are present at the RF and IF stages of the mixer as shown in Figure 8. Moreover, Figure 9 shows the design of first-order bandpass filter present at the source terminals of the LOI stage transistors at one end and the power supply at the other end to avoid leakage from the power supply.

The input and output impedance of the ninth-order filter section can be expressed as a combination of series and parallel LC sections within the design. The filter order depends on the number of LC pairs. The input impedance, *Z*_in_ is expressed as:

(40) $$Z_{\mathrm{in}}=[[[[[(sL_{1}\left|\right|\frac{1}{sC_{1}}+sL_{2}\left|\right|\frac{1}{sC_{2}})||\frac{1}{sC_{3}}]+sL_{3}\left|\right|\frac{1}{sC_{4}}]||sC_{5}]+sL_{4}\left|\right|\frac{1}{sC_{6}}]||\frac{1}{sC_{7}}]+sL_{5}\left|\right|\frac{1}{sC_{8}}]||sL_{6}\left|\right|\frac{1}{sC_{9}}]$$

(41) $$Z_{\mathrm{in}}=\left(Y+F\right)\left|\right|G$$

(42) Y=(X+D)||E

(43) X=(A+B)||C

(44) $$A=(sL_{1}\left|\right|\frac{1}{sC_{1}}+sL_{2}\left|\right|\frac{1}{sC_{2}})||\frac{1}{sC_{3}}$$

(45) $$B=sL_{3}\left|\right|\frac{1}{sC_{4}}=\frac{sL_{3}}{s^{2}L_{3}C_{4}+1}$$

(46) $$C=\frac{1}{sC_{5}}$$

(47) $$D=sL_{4}\left|\right|\frac{1}{sC_{6}}=\frac{sL_{4}}{s^{2}L_{4}C_{6}+1}$$

(48) $$E=\frac{1}{sC_{7}}$$

(49) $$F=sL_{5}\left|\right|\frac{1}{sC_{8}}=\frac{sL_{5}}{s^{2}L_{5}C_{8}+1}$$

(50) $$G=sL_{6}\left|\right|\frac{1}{sC_{9}}=\frac{sL_{6}}{s^{2}L_{6}C_{9}+1}$$

For simplicity, different letters have been used to represent the LC combinations. The output impedance, *Z*_out_ is expressed as:

(51) $$Z_{\mathrm{out}}=sL_{6}\left|\right|\frac{1}{sC_{9}}$$

Similarly, the first-order impedance depends on the parallel combination of L and C.

(52) $$Z_{P}=Z_{L}\left|\right|Z_{C}=\frac{Z_{L}Z_{C}}{Z_{L}+Z_{C}}$$

The resonant frequency at which the impedance, *Z*_P_ will be real can be defined as

(53) $$f_{0}=\frac{1}{2\pi \sqrt{L_{1}C_{1}}}$$

or

(54) $$\frac{1}{2\pi \sqrt{L_{2}C_{2}}}$$

where *Z*_P_ refers to the parallel circuit impedance. The resonant frequency varies depending on the selected filter circuit within the design.

## 4. Mixer Design Analysis

Figure 4 shows the complete structure of the designed mixer with *g*_m_-boosting, common source configured transconductance stage and Gilbert cell core stage. The first step is to determine the band of operation. The next step is choosing the design topology and filters for successful reconfiguration. The design uses Gilbert topology responsible for improving the overall CG, NF. To further enhance this performance, *g*_m_-boosting with inductive peaking is employed. The design is structured to provide good image rejection as well without affecting performance of the design.

### 4.1. Conversion Gain

**Figure 10:**
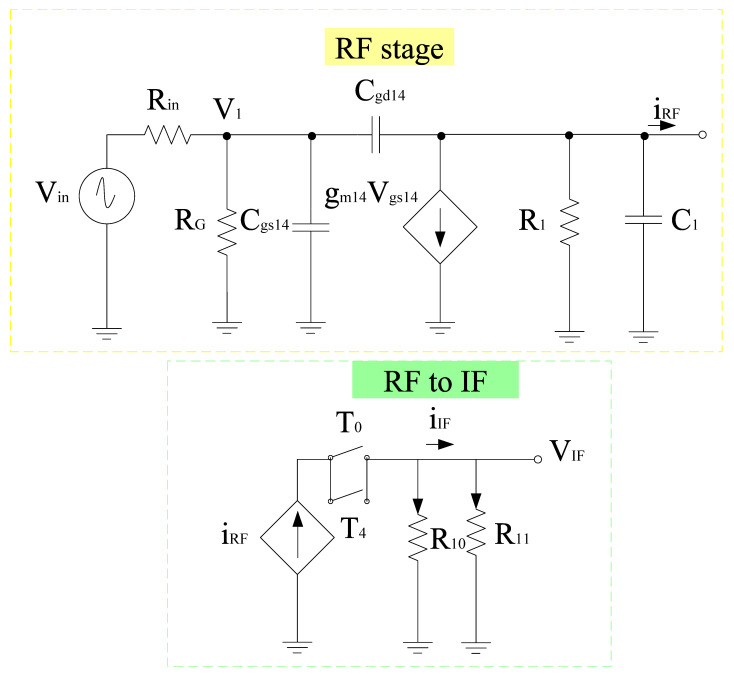
Complete small signal model.

Figure 10 shows the complete small signal model used for obtaining the overall CG within the design. The CG, *A*_v_ is represented by the expression below:

(55) $$A_{v}=\frac{V_{\mathrm{IF}}\left(s_{\mathrm{IF}}\right)}{i_{\mathrm{IF}}\left(s_{\mathrm{IF}}\right)}\frac{i_{\mathrm{IF}}\left(s_{\mathrm{IF}}\right)}{i_{\mathrm{RF}}\left(s_{\mathrm{RF}}\right)}\frac{i_{\mathrm{RF}}\left(s_{\mathrm{RF}}\right)}{V_{\mathrm{gs}14}\left(s_{\mathrm{RF}}\right)}\frac{V_{\mathrm{gs}14}\left(s_{\mathrm{RF}}\right)}{V_{\mathrm{in}}\left(s_{\mathrm{RF}}\right)}$$

where all expressions in Equation (55) are obtained using the small signal model except *i*_IF_(*s*_IF_)/*i*_RF_(*s*_RF_) which can be obtained using Fourier series analysis by approximating LO signal just like a square wave.

(56) $$\frac{V_{\mathrm{gs}14}\left(s_{\mathrm{RF}}\right)}{V_{\mathrm{in}}\left(s_{\mathrm{RF}}\right)}=\frac{1}{\left(1+\frac{R_{\mathrm{in}}}{R_{G}}\right)+sC_{\mathrm{gs}14}R_{\mathrm{in}}}$$

(57) $$\frac{V_{\mathrm{IF}}\left(s_{\mathrm{IF}}\right)}{i_{\mathrm{IF}}\left(s_{\mathrm{IF}}\right)}=\frac{R_{10}R_{11}}{R_{10}+R_{11}}$$

(58) $$\frac{i_{\mathrm{IF}}\left(s_{\mathrm{IF}}\right)}{i_{\mathrm{RF}}\left(s_{\mathrm{RF}}\right)}=\frac{2}{\pi }$$

For determining *i*_RF_(*s*_RF_)/*V*_gs14_(*s*_RF_) ratio KCL is applied, and we obtain the expression below:

(59) $$V_{\mathrm{gs}14}\left(s_{\mathrm{RF}}\right)[s\left(C_{\mathrm{gs}14}+C_{\mathrm{gd}14}\right]=g_{m14}V_{\mathrm{gs}14}+i_{\mathrm{RF}}\left(s_{\mathrm{RF}}\right)\left[\frac{1}{R_{1}}+sC_{1}\right]$$

Rearranging the above equation, we get;

(60) $$V_{\mathrm{gs}14}\left(s_{\mathrm{RF}}\right)\left[sC_{\mathrm{gs}14}+sC_{\mathrm{gd}14}-g_{m14}\right]=i_{\mathrm{RF}}\left(s_{\mathrm{RF}}\right)\left(\frac{1}{R_{1}}+sC_{1}\right)$$

(61) $$\frac{i_{\mathrm{RF}}\left(s_{\mathrm{RF}}\right)}{V_{\mathrm{gs}14}\left(s_{\mathrm{RF}}\right)}=\frac{sC_{\mathrm{gs}14}+sC_{\mathrm{gd}14}-g_{m14}}{\frac{1}{R_{1}}+sC_{1}}$$

By substituting (56)–(61) into (55), the overall CG can be obtained.

### 4.2. Noise Figure

**Figure 11:**
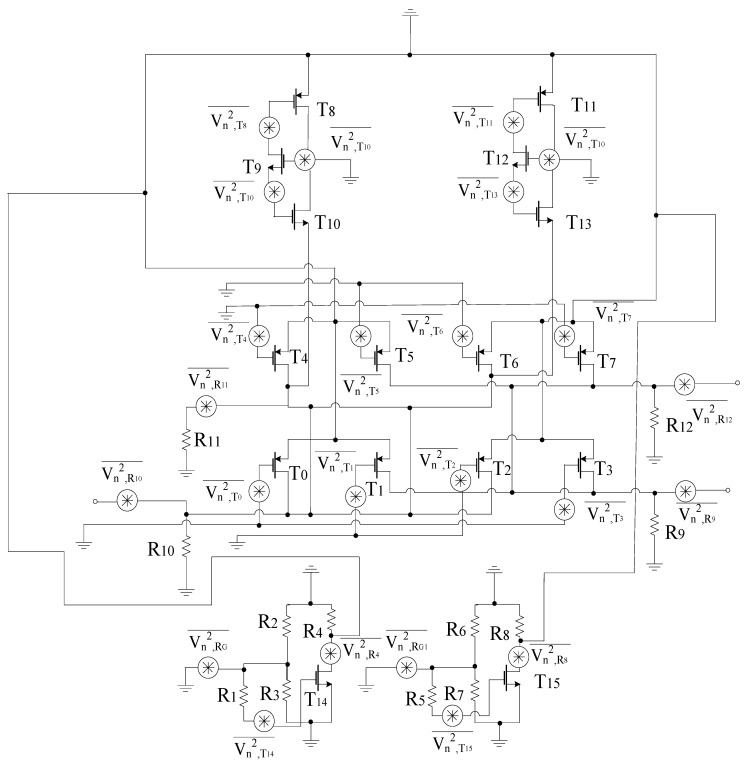
Proposed mixer noise model.

Figure 11 shows the noise model for the proposed circuit. In the circuitry, all passive elements are considered ideal, and the most important noise source is thermal noise, based on which the power spectral density of each stage is obtained. The design consists of resistors and transistors, respectively [41,46].

Equation (62) defines the power spectral density, which is the combination of the power spectral density obtained from all stages present within the design.

(62) $$\bar{V^{2}{}_{n}}=\bar{V^{2}{}_{n,RF}}+\bar{V^{2}{}_{n,LOI}}+\bar{V^{2}{}_{n,LOQ}}+\bar{V^{2}{}_{n,GM}}$$

The power spectral density for all stages is obtained based on the resistors and transistors present within each stage. Thus, the power spectral density for the RF+ stage is expressed as

(63) $$\bar{V^{2}{}_{n,RF+}}=\bar{V^{2}{}_{n,R_{1}}}+\bar{V^{2}{}_{n,R_{2}}}+\bar{V^{2}{}_{n,R_{3}}}+\bar{V^{2}{}_{n,R_{4}}}+\bar{V^{2}{}_{n,T_{14}}}=\frac{4kT\gamma }{g_{m14}}+4kTR_{3}+4kTR_{2}+4kTR_{4}+4kTR_{1}$$

Likewise, the power spectral density for RF- stage is defined below

(64) $$\bar{V^{2}{}_{n,RF-}}=\bar{V^{2}{}_{n,R_{5}}}+\bar{V^{2}{}_{n,R_{6}}}+\bar{V^{2}{}_{n,R_{7}}}+\bar{V^{2}{}_{n,R_{8}}}+\bar{V^{2}{}_{n,T_{15}}}=\frac{4kT\gamma }{g_{m15}}+4kTR_{5}+4kTR_{6}+4kTR_{7}+4kTR_{8}$$

Moreover, the power spectral densities for LOI and LOQ stages are expressed as

(65) $$\bar{V^{2}{}_{n,LOI}}=\bar{V^{2}{}_{n,T_{4}}}+\bar{V^{2}{}_{n,T_{5}}}+\bar{V^{2}{}_{n,T_{6}}}+\bar{V^{2}{}_{n,T_{7}}}+\bar{V^{2}{}_{n,R_{11}}}+\bar{V^{2}{}_{n,R_{12}}}$$

(66) $$\bar{V^{2}{}_{n,LOI}}=4kTR_{11}+4kTR_{12}+\frac{4kT\gamma }{g_{m4}}+\frac{4kT\gamma }{g_{m5}}+\frac{4kT\gamma }{g_{m6}}+\frac{4kT\gamma }{g_{m7}}$$

(67) $$\bar{V^{2}{}_{n,LOQ}}=\bar{V^{2}{}_{n,T_{0}}}+\bar{V^{2}{}_{n,T_{1}}}+\bar{V^{2}{}_{n,T_{2}}}+\bar{V^{2}{}_{n,T_{3}}}+\bar{V^{2}{}_{n,R_{9}}}+\bar{V^{2}{}_{n,R_{10}}}$$

(68) $$\bar{V^{2}{}_{n,LOQ}}=4kTR_{9}+4kTR_{10}+\frac{4kT\gamma }{g_{m0}}+\frac{4kT\gamma }{g_{m1}}+\frac{4kT\gamma }{g_{m2}}+\frac{4kT\gamma }{g_{m3}}$$

(69) $$\bar{V^{2}{}_{n,GM}}=\bar{V^{2}{}_{n,GM+}}+\bar{V^{2}{}_{n,GM-}}$$

Finally, the power spectral densities for GM stages are expressed as

(70) $$\bar{V^{2}{}_{n,GM+}}=\bar{V^{2}{}_{n,T_{8}}}+\bar{V^{2}{}_{n,T_{9}}}+\bar{V^{2}{}_{n,T_{10}}}=\frac{4kT\gamma }{g_{m9}}+\frac{4kT\gamma }{g_{m8}}+\frac{4kT\gamma }{g_{m10}}$$

(71) $$\bar{V^{2}{}_{n,GM-}}=\bar{V^{2}{}_{n,T_{11}}}+\bar{V^{2}{}_{n,T_{12}}}+\bar{V^{2}{}_{n,T_{13}}}=\frac{4kT\gamma }{g_{m11}}+\frac{4kT\gamma }{g_{m12}}+\frac{4kT\gamma }{g_{m13}}$$

Hence, the noise figure of the proposed mixer is expressed as

(72) $$NF=1+\frac{\bar{{V^{2}}_{n}}}{{A_{v}}^{2}}$$

Moreover, for analyzing the high-frequency noise in the proposed mixer, the thermal noise due to resistors, the thermal noise due to drain, and gate of FETs are considered [47]. The noise contribution due to RF, LOI, LOQ and output stages are considered for the proposed mixer.

**Figure 12:**
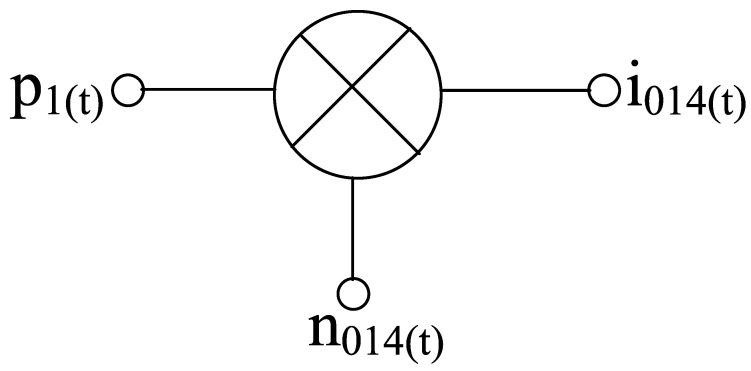
Mixer operation for transconductance noise.

Starting with the noise contribution from the RF stage as per Figure 12, the noise signal at the output of the transconductor when multiplied with the switching pair’s instantaneous current gain *p*_1(t)_ results in a current noise , *i*_o14(t)_ as

(73) $$i_{o14(t)}=n_{o14(t)}p_{1(t)}$$

By considering the above process as a time-average wide sense stationary process, the power spectral density of the noise current is expressed as

(74) $$<S^{0}{}_{n014}\left(\omega t\right)>\;=\sum_{n=-\infty }^{\infty }{\left|p_{1,n}\right|}^{2}S_{n014}\left(\omega -n\omega_{LO}\right)$$

For the overall analysis of power spectral density at the RF stage, both correlated and uncorrelated power spectral density factors have to be considered. Thus, the uncorrelated power spectral density is expressed as

(75) $$S^{\left(u\right)}{}_{n014}(\omega ){|}_{\bar{V^{2}{}_{n,rg014}}+\bar{V^{2}{}_{n,RG014}}}=\left(\bar{V^{2}{}_{n,rg014}}+\bar{V^{2}{}_{n,RG014}}\right)\frac{g^{2}{}_{m14}}{{\left[\omega \left(C_{gs14}+C_{gd14}\right)R_{GG014}\right]}^{2}+1}$$

where *R*_G014_, *r*_g014_ refer to the external gate resistance (parallel combination of the resistors) and internal gate resistance.

(76) $$S^{\left(u\right)}{}_{n014}(\omega ){|}_{i_{ng014,u}}\approx \bar{I^{2}{}_{ng014,u}}\frac{g^{2}{}_{m14}}{{\left[\omega \left(C_{gs14}+C_{gd14}\right)R_{GG014}\right]}^{2}+1}$$

Likewise, the correlated power spectral density can be expressed as

(77) $$S^{\left(c\right)}{}_{n014}(\omega )=\left[{\left(k_{c}+1\right)}^{2}+k^{2}{}_{c}{\left|H_{T}14\left(\omega \right)\right|}^{2}-2k_{c}\left(k_{c}+1\right)Re\left[H_{T14}\left(\omega \right)\right]\right]\bar{I^{2}{}_{nd014}}$$

Thus, the overall power spectral density is expressed as

(78) $$S_{n014}\left(\omega \right)=S^{\left(u\right)}{}_{n014}(\omega )+S^{\left(c\right)}{}_{n014}(\omega )={\left(k_{c}+1\right)}^{2}A_{s014}\bar{I^{2}{}_{nd014}}\left[\frac{1+\frac{1}{A_{s014}}{\left(\frac{\omega }{\omega_{z014}}\right)}^{2}}{1+{\left(\frac{\omega }{\omega p014}\right)}^{2}}\right]$$

Next, the power spectral density due to LO stages is expressed as

(79) $$\begin{array}{c} S_{n01}\left(\omega \right)+S_{n45}\left(\omega \right)=2\left[S^{\left(u\right)}{}_{n01}(\omega )+S^{\left(c\right)}{}_{n01}(\omega )+S^{\left(u\right)}{}_{n45}(\omega )+S^{\left(c\right)}{}_{n45}(\omega )\right]=\\ 2[{\left(k_{c}+1\right)}^{2}As_{01}\bar{I^{2}{}_{nd01}}\left[\frac{1+\frac{1}{A_{s01}\left({\frac{\omega }{\omega_{z01}}}^{2}\right)}}{1+({\frac{\omega }{\omega_{p01}}}^{2})}\right]+{\left(k_{c}+1\right)}^{2}As_{45}\bar{I^{2}{}_{nd45}}\left[\frac{1+\frac{1}{A_{s45}\left({\frac{\omega }{\omega_{z45}}}^{2}\right)}}{1+({\frac{\omega }{\omega_{p45}}}^{2})}\right]] \end{array}$$

Considering the noise present at LO ports are stationary. Thus, time-averaged power spectral density of current noise at the output of the proposed mixer due to LO stages is expressed as

(80) $$<S^{0}{}_{nLO}(\omega t)>=4kT\left(R_{LOI}+2r_{G1}\right)\bar{G^{2}}\left(t\right)+4kT\left(R_{LOQ}+2r_{G2}\right)\bar{G^{2}}\left(t\right)$$

where *R*_LOI_, *R*_LOQ_ refer to equivalent noise resistances and *r*_G1_, *r*_G2_ refer to poly gate resistances. As the image signal does not carry any important information, therefore the single sideband noise figure is considered over the double sideband noise figure as

(81) $$\begin{array}{c} NF_{\mathrm{SSB}}=\frac{\int_{0}^{\infty }<S^{0}{}_{n014}(\omega ,t)>d\omega }{|g_{c}{\left(\omega \right)|}^{2}}\frac{1}{4kTR_{G}}\\ =\frac{<S^{0}{}_{n014}(\omega ,t)>+S^{0}{}_{n01}(\omega ,t)+S^{0}{}_{n45}(\omega ,t)+<S^{0}{}_{nLO}(\omega ,t)>+(4kTR_{10}+4kTR_{11}\left|\right|G_{M})}{|g_{c}{\left(\omega \right)|}^{2}} \end{array}$$

The above expression is defined for a single balanced mixer. Similarly, for the double balanced mixer, NF can also be defined which is almost twice the one obtained for a single balanced mixer.

## 5. Results and Discussion

**Figure 13:**
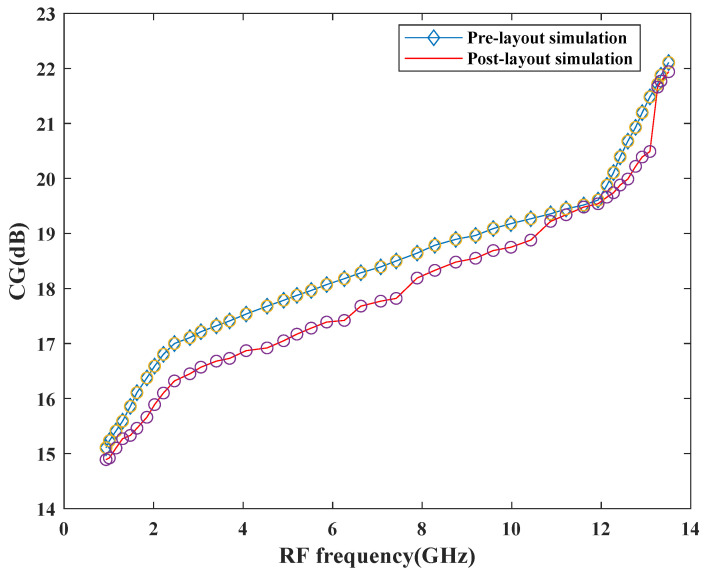
Variation of CG versus frequency.

The proposed mixer is designed and simulated in the SiGe 8HP process technology. To boost the transconductance within the RF stage, *g*_m_-boosting technique has been used in the design, which leads to good CG performance. Figure 13 shows the pre-and post-layout simulation results for the conversion gain performance of the proposed mixer. As depicted in Figure 13, the CGmin and CGmax values are quite similar for both simulations. However, variation can be observed at other frequencies within a band that can be discussed by considering the parasitic effects. The pre-layout CG at the center frequency, 7 GHz is 18.39 dB and after layout, it degrades to 17.7 dB. Due to the parasitic effects of passive components within the circuitry, the CG degrades after the final layout. In particular, the quality factors of the inductors within the circuitry are responsible for the gain performance degradation. Moreover, the parasitic resistance within the inductors can also lower the voltage gain within the circuitry.

**Figure 14:**
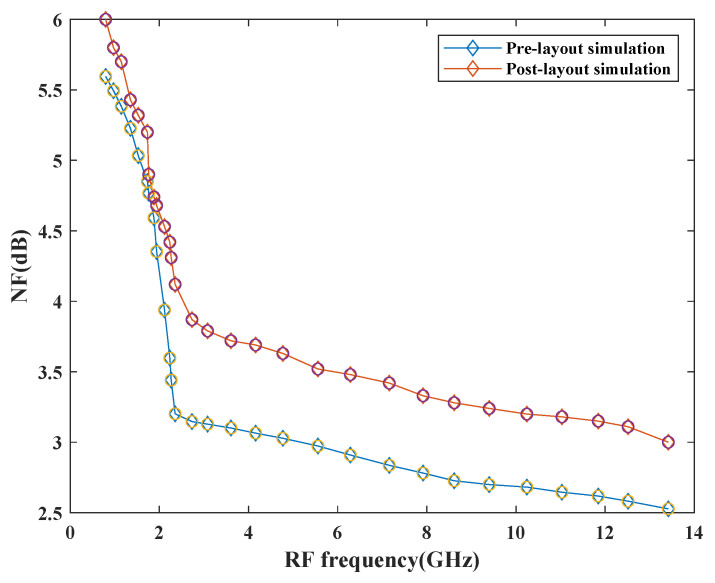
Variation of NF with RF frequency.

Figure 14 depicts the NF of the mixer with the variation in frequency. The simulation results show that NF is less than 3 dB before pre-layout simulation and raised by 0.8 dB upon post-layout simulation at the maximum frequency. This performance has also been affected by the parasitic effects of passive components. It also has dependency on the number of resistive components, transistors, and conversion gain performance of the design. This mixer exhibits good NF with a variation of ±1 dB across the entire frequency range.

**Figure 15:**
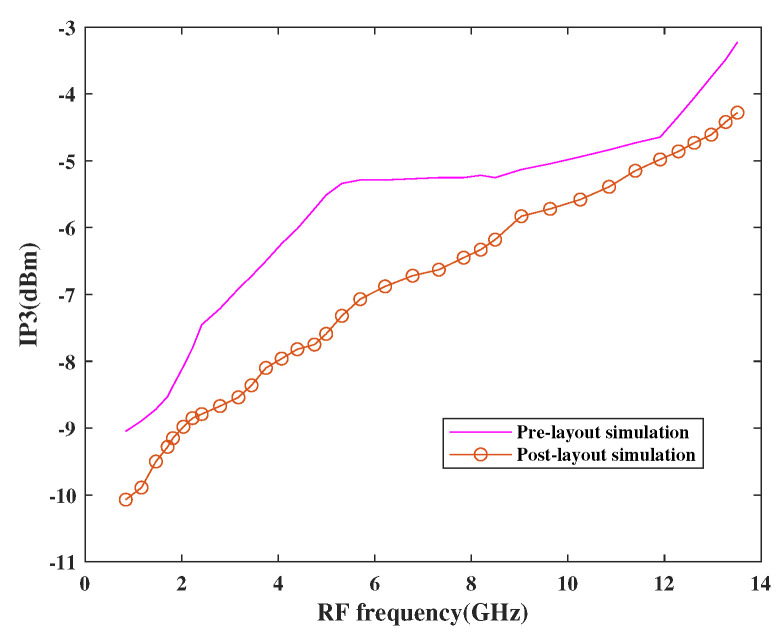
Variation of IP3 with RF frequency.

**Figure 16:**
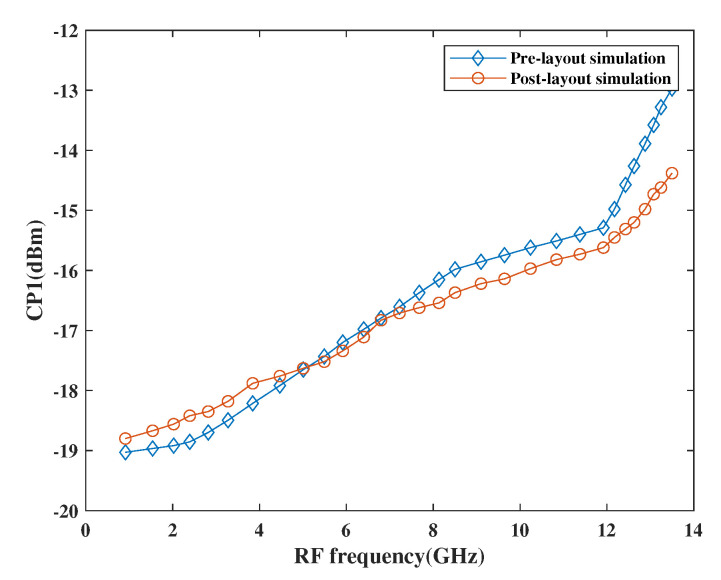
Variation of CP1 with RF frequency.

The linearity performance of the mixer has been shown in Figure 15 and Figure 16, respectively. The design is considered linear if it shows proportional behavior within the input and output. This behavior can be observed using third-order input intercept points (IP3) and 1dB compression point (CP1). The actual behavior of the mixer is well depicted in terms of pre-and post-layout simulation results. As per the simulation results, it has been observed that the design attained moderate linearity behavior when observed at different frequencies in a band where IP3 is 10 dBm higher than CP1.

**Figure 17:**
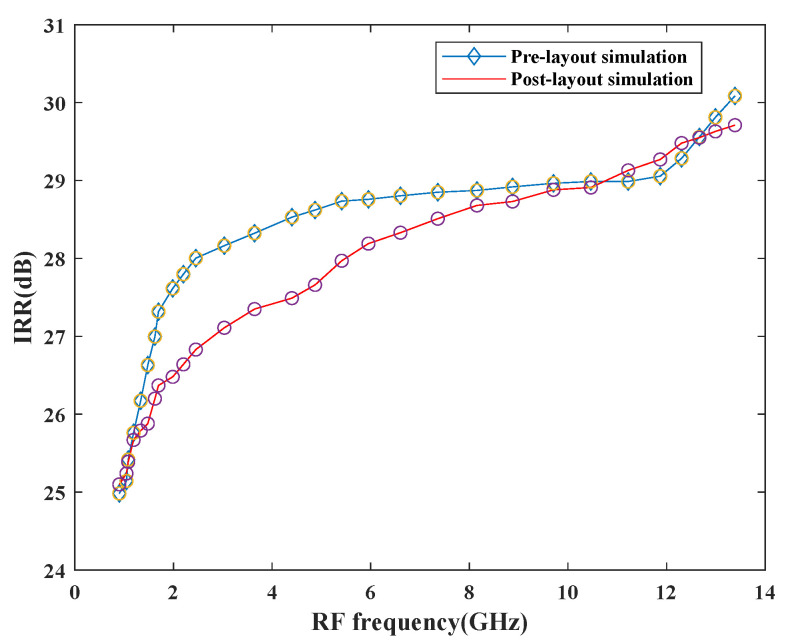
Variation of IRR with RF frequency.

The image-rejection ratio is an important aspect while designing the mixer as depicted in Figure 17. When desired and image signals enter the input together, it degrades the overall performance of the circuitry and waste power. Therefore, to overcome this problem, the image signal must be rejected which is done in the proposed design. The mixer attains a good IRR of 28.91 dB at 10.46 GHz upon performing pre- and post-layout simulations. The maximum IRR is around 30 dB, which is within the normal specified IRR range of 20–40 dB.

**Figure 18:**
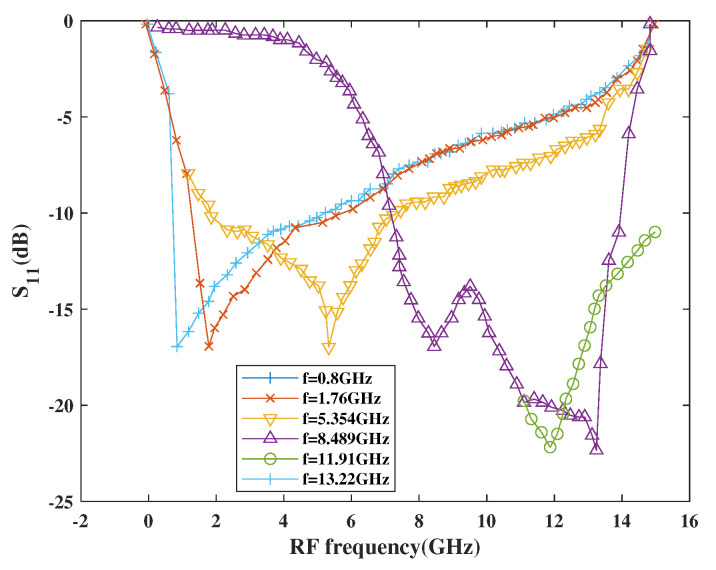
Variation of S_11_ with RF frequency.

Figure 18 shows the return loss performance with respect to frequency. As per simulation results, the |S_11_| is below 10 dB at each centered frequency for the entire tuning band, which is as low as −22.42 dB at 11.91 GHz and 13.22 GHz, respectively.

**Figure 19:**
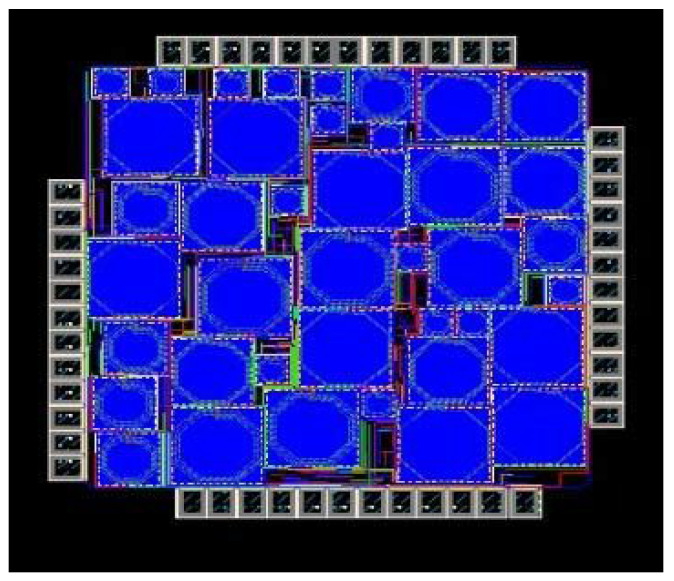
Designed Mixer Layout.

Figure 19 shows the layout of the proposed mixer designed in 8 HP process technology covering around 1.98 mm^2^ area. The design consists of different sections as discussed in detail in Section 3. The filter section consists of spiral inductors and capacitors. Inductors used provide accurate inductance values and are capable of achieving the maximum Q at a desired operating frequency. Additionally, variable capacitors, i.e., varactors, are used to attain the tuning capacitance.

Table 1 summarizes the performance of the proposed mixer and provides a comparison of the circuit with the recent works. As per Table 1, the achievable NF is as less as 2.5 dB and the maximum S_11_ is −20 dB. As CG increases, the IP3 gets degraded due to CG-IP3 trade-off. Moreover, the maximum IRR is 36 dB. The overall area of the proposed mixer is higher than the other reported designs. However, the design attains high performance in terms of CG, NF, IRR and S_11_ simultaneously at the expense of IP3 which is the best among all reported works in the literature.

## 6. Design Reliability

**Figure 20:**
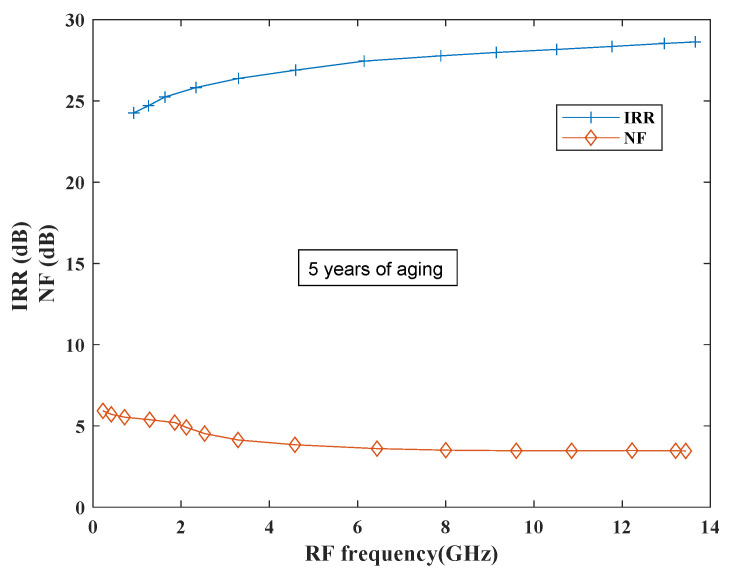
IRR and NF degradation performance.

Conventional designs were less focused on reliability analysis due to the process and design guide limitations. However, in recent years it is important to consider reliability of the designs due to time, budget, scaling and demanding profile constraints. Relxpert tool developed by Cadence is used to simulate PFET and NFET devices for determining the device degradation performance where performance is evaluated as a function of stress time and biases. Relexpert output can be observed as a “corner in time” that moves towards slow corners in case of simulation from a typical corner. This process helps the designers in analyzing the degradation in circuit behavior during the initial design flow stages [58]. Degradation performance has been evaluated in terms of all performance parameters such as CG, CP1, IP3, NF and IRR ratio. Figure 20 shows the performance of the proposed mixer in terms of IRR and NF with 5 years of aging. From the plots it has been observed that both IRR and NF show degradation; however, more degradation can be observed in NF in comparison to IRR.

**Figure 21:**
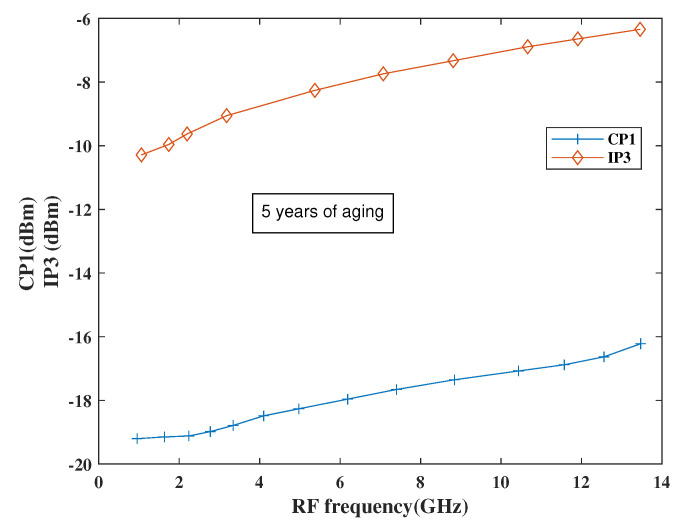
CP1 and IP3 degradation performance.

Figure 21 shows the performance of the proposed mixer in terms of linearity parameters and it has been found that the linearity will degrade as expected as such variation is observed after post-layout simulation results as well. However, the NF is still ≤ 5 dB within the entire band, which is expected for a mixer.

**Figure 22:**
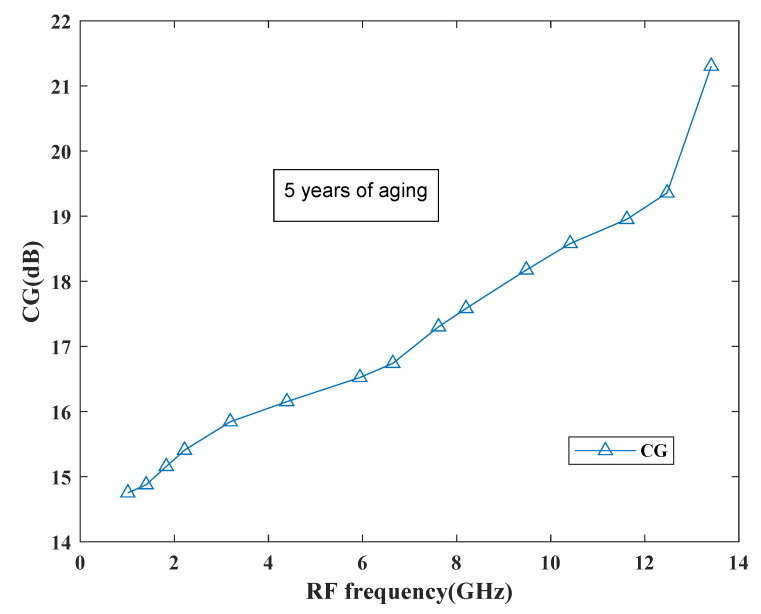
CG degradation performance.

The behavior of the gain can be observed from Figure 22 which shows the performance of the proposed mixer in terms of gain. Based on the curve, it has been found that the gain degradation is very less as it reaches to 21.5 dB after 5 years and at present it is around 22.1 dB. Thus, the proposed mixer is reliable for future SDR applications.

## 7. Conclusions

In this paper, a novel reconfigurable I/Q Gilbert mixer has been proposed, which is designed and simulated in SiGe 8HP process technology. ninth-order tunable LC filters are embedded at the RF and IF ports for port matching and NF improvement. Moreover, a first-order tunable filter is employed to avoid the leakage through the power supply. The proposed design shows improved transconductance by using G_m_-boosting technique. Additionally, the employment of peaking inductors compensates for the gain reduction at high frequencies, while extending the overall bandwidth and hence results in a high gain. Based on the simulation results, with a 1.2 V power supply, the design attains a maximum gain of 22.1 dB. The input return loss is <−10 dB and achieves a minimum of −22.7 dB at 11.9 GHz and 13.22 GHz, respectively. Furthermore, NF ranges between 2.5 and 5.6 dB. The design also shows good IRR within the entire band. Thus, the proposed mixer is compatible enough to meet the future demands of software-defined radios. 

## Figures and Tables

**Table 1:** Performance Comparison Summary.

Ref.	Tech.	Area (mm^2^)	Freq. (GHz)	S_21_ (dB)	NF (dB)	IRR (dB)	IP_3_ (dBm)	S_11_ (dB)
This work	SiGe 8HP	1.8	0.9–13.5	15.1–22.1	2.5–5.6	24.9–30.2	−3.28–9.05	−17.14–22.7
[42]	0.25 um	Nil	0.9	5	8	30	1	−15
[8]	0.065 um	0.19	0.9, 1.8–2.5	9.2–13	13.6–18.3	Nil	≥10.8	Nil
[48]	0.18 um	Nil	2.42–2.48	10.73	Nil	Nil	−7.31	Nil
[49]	0.18 um	Nil	2.4	9.3	7.4	Nil	8	Nil
[26]	0.18 um	Nil	2.4	17	11	Nil	1	Nil
[34]	0.18 um	Nil	2.44	18.6	7.15	Nil	−8.1	Nil
[50]	0.18 um	<1	3.1–10.6	≥10	10	Nil	4	−25
[5]	0.18 um	1.4	5.1	18	13.2	Nil	−5.85	−14.5
[44]	SiGe	0.9	5.1–5.8	14	6.8	36	−5.5	−11
[32]	0.13 um	0.85	7.2–8.4	23.8	4.3	30	−10.5	Nil
[51]	0.18 um	0.11	1.8–2.4	23–26	16–20	Nil	−2	Nil
[52]	0.18 um	0.61	0.5–7.5	5.7	15	Nil	−5.7	Nil
[53]	0.18 um	1.14	3–5	19.8–20.6	7.7–8.7	Nil	>−6	−10.5–15.2
[25]	0.065 um	0.21	1–10.5	10–14.5	6.5–10	Nil	Nil	−20
[54]	0.13 um	0.31	1–5.5	17.5	3.9	Nil	0.84	<−8.8
[55]	0.09 um	0.57	80–110	4.1–11.6	15.8–18.1	Nil	3	−8.7–22
[56]	0.065 um	0.5	17–43	−0.1 ± 1.5	12.4	Nil	3.4	Nil
[57]	0.13 um	0.13	0.87–3.7	13.5–14	2.9–6.5	Nil	−10–13	Nil

## Data Availability

Not applicable.
